# Evidence That Sleep Is an Indicator of Overtraining during the Competition Phase of Adolescent Sprinters

**DOI:** 10.1155/2021/6694547

**Published:** 2021-04-03

**Authors:** Eon H. Campbell, Melanie Poudevigne, Shelly McFarlane, Lowell Dilworth, Rachael Irving

**Affiliations:** ^1^Department of Basic Medical Sciences, The University of the West Indies, Mona, Jamaica; ^2^Health and Fitness Management, Clayton State University, Morrow, GA, USA; ^3^Caribbean Institute for Health Research, The University of the West Indies, Mona, Jamaica; ^4^Department of Pathology, The University of the West Indies, Mona, Jamaica

## Abstract

Although sleep disturbance is a common complaint in overtrained athletes, the role of sleep in the overtraining process is not clear. This study aimed (i) to compare sleep efficiency/quantity at the start of a competition phase in elite adolescent sprinters who adapted to prior training with that in those who maladapt and (ii) to examine the influence of prior training, fatigue, and sleep on performance through a moderated mediation model. Fatigue (via Profile of Mood State) and internal training load (via session rating of perceived exertion and duration of training as volume) were measured in 20 sprinters (mean age: 15.9 ± 1.7 years) across 4 mesocycles (baseline (T1); preparatory (T2); precompetitive (T3); and competitive (T4) phases), over 26 weeks. Performances were assessed during the competitive period (T3, T4), while sleep was monitored (via actigraphy) for a week during T4. It was inferred that sprinters who had increasingly greater fatigue and concomitant decrements in performance (35%) were maladapted to training and the remaining sprinters who improved fatigue and performance (65%) were adapted to training. Sleep efficiency (91 ± 3% vs. 82 ± 3%, *p* < 0.001) and quantity (425 ± 33 min vs. 394 ± 20 min, *p* < 0.001) at the start of T4 were significantly greater in sprinters who adapted. Moreover, higher prior training volume (mean of T1 to T3 training volume) was associated with lower sleep efficiency at the start of T4 (*R*^2^ = 0.55, *p* < 0.001) which was associated with poorer performance (*R*^2^ = 0.82, *p* < 0.001). Fatigue moderated the indirect effect of prior training volume on performance through its moderation of the effect of sleep efficiency on performance (*R*^2^ = 0.89, *p* < 0.001). Impaired sleep as a result of greater prior training volume may be related to performance decrements through fatigue. Athletes should improve sleep during periods of higher training volume to reduce fatigue for better adaptation to training.

## 1. Introduction

Sleep has been recognized as an essential component in athletic preparation and performance and is considered to be one of the best recovery strategies available to athletes [1]. The risk of underrecovery is possible in athlete's preparation, as periods of high intensity training are used to push athletes beyond the very limit of their physical capacity, thereby entering a state typically referred to as functional overreaching (FOR). Many researchers consider this a desirable phase in training regimens, done in an attempt to enhance physiological adaptation and improve performance [2]. However, if prolonged and not balanced with adequate recovery, FOR could lead to nonfunctional overreaching (NFOR) and eventually to a more severe maladaptation, termed the overtraining syndrome (OTS) [2, 3]. The overtraining process is on a continuum from FOR to OTS, to distinguish between short-term (acute) decrement in performance followed by supercompensation (improvement in performance) after recovery and long-term (chronic) decrements in performance capacity where allied psychophysiological symptoms are seen [2–4].

There is evidence that sleep disturbance occurs in the overtraining process, but the majority of this evidence is in endurance activities with prevalence rates as high as 60% [2, 5, 6]. These studies showed that in athletes who suffered from overreaching, sleep efficiency and quantity measured during periods of overloading (high volume and intensity training) were significantly less than those in nonoverreached athletes [5, 7, 8]. Currently, there are some deficiencies in the literature regarding the prevalence of overtraining among athletes involved in anaerobic activities such as sprint, in opposition to athletes involved in endurance events. A previous study, however, has proposed that overtraining in anaerobic exercise can elicit considerably different biological responses when compared to overtraining in endurance activities [2]. The mechanisms behind these contrasting responses have not yet been established, and it is currently unclear whether sleep disturbance is an etiological mechanism of overreaching or merely a symptom. Many mechanisms have been shown to contribute to the impact of sleep disturbances on athletic performance [9]. The literature has shown a detrimental effect of sleep deprivation and a beneficial effect of chronic sleep improvement on sport-specific and physical performance [10, 11], yet the effect of sleep in overreaching is unclear [9]. Notwithstanding, a growing body of evidence has confirmed the link between critical sleep factors, and several pathways and responses which modulate the overtraining process [9]. For example, research has confirmed that sleep extension and deprivation have respective positive and negative effects on mood, fatigue, and cognitive functions [12, 13]. Furthermore, it has been established that during periods of sleep deprivation many markers of innate and adaptive immunity are depressed and immune function decreases [9]. Studies also supported the assumption of the critical effects of sleep on neuroendocrine and metabolic responses [14, 15]. These responses have previously been studied in overtraining affected individuals [2–4] and have been confirmed as markers in identifying OTS [2, 4, 15]. However, the links between these differential aspects, in sleep deprived versus overreached athletes, are currently not fully understood.

A number of potential factors that may contribute, specifically, to inadequate sleep in overreached athletes have surfaced in the literature. Two of these factors, accompanying each other, are training load (volume and intensity) and timetabled environment for adolescents who have early start time classes, followed by evening training and competition [16, 17]. In fact, increased training loads have been negatively associated with sleep efficiency/quantity in athletes [8]. Using a study conducted in triathletes, for example, Hausswirth and colleagues found decreases in sleep efficiency/quantity following an augmented increase (+30%) in training load and reductions to baseline value during the ensuing taper [8]. Increases in training load are associated with compromised immune, muscular, neuroendocrine, and psychological responses, which appear to occur in a dose-dependent manner [9, 14, 15, 18]. This means that as training load increases, so do mood disturbance and fatigue, with mood and fatigue improving once training loads are reduced. In adolescents, academic induced sleep deficit can be augmented by periods of greater training loads to further potentiate these responses, negatively affecting performance [17]. While this interrelation is known, it is unclear whether the relation is moderated by other factors such as fatigue or mood state [19].

In the present study, we investigated whether adolescent athletes who “adapt” to prior training cycle (i.e., athletes who maintained or improved fatigue and performance) sleep efficiency and quantity during the competitive phase would be different from athletes who “maladapt” (i.e., athletes who had concomitantly high perceived fatigue and declined performance). We then examined, through cross-sectional analyses, the relations between prior training, psychophysiological factors, relative change in performance, and sleep measured during the start of the competition phase, to suggest possible pathways and mechanisms linking the said variables through moderated mediation analyses. This was done as it is important to understand the associations between training, sleep, and performance in athletes so as to provide practical implication to combat training maladaptation.

The present study offers new contributions. First, the majority of research examining sleep in the overtraining processes focused on endurance athletes [1, 8], with limited research on sleep, in NFOR/OTS athletes who participate in sprinting events [6]. The current study builds on these studies by examining sleep in sprint athletes around an ecological training program, which allowed for a direct comparison of sleep in athletes who adapted to a training cycle with those who maladapt. Additionally, previous studies that used sleep to identify athletes at risk of training maladaptation were conducted mainly in adults. Special consideration should be given to younger athletes, as adolescents in particular exhibit a higher physiological need for sleep and experience delayed timing of sleep onset and awakening due to increased academic demands, social desires, and early school start time [17, 20]. Second, the prevalence rates (15%–70%) of sleep inadequacy have been reported to be high among elite adult athletes [18]. These athletes often experience disruptive training and competition schedules that limit the opportunity for sleep [18], but do we know whether or not this is also the case for the adolescent athletes in timetabled educational environments with training in the evenings? Third, although studies have previously found that training loads (intensity x volume) directly influence sleep efficiency in athletes [5, 8, 16], no studies have explored whether the particular aspects of training load for the purpose of sprinting (volume and/or intensity) are more related to sleep disturbances [1] or have explored the possible link among common symptoms associated with overtraining such as sleep, fatigue, mood state, and performance decline. Thus, the goals of the present study were to evaluate these understudied aspects and to better understand training maladaptation in adolescent athletes.

In the present study, we hypothesized (H1) that athletes who adapted to the prior training program would have significantly better sleep efficiency and quantity at the start of competition phase than athletes who maladapt to the prior training program. This prediction is in line with studies which found that sleep efficiency and quantity occurred in parallel with NFOR/OTS in athletes [1]. Considering that (i) increases in training loads are needed to promote physiological adaptation, (ii) sleep is a vital component of both mental and physical recovery from exercise, and (iii) excessive training loads and sleep disturbance may potentiate fatigue, we hypothesized (H2) that sleep efficiency/quantity at the beginning of the competition phase would be positively related to performance in adolescent sprinters, while prior internal training loads/volume and fatigue would be negatively related to performance. Finally, we hypothesized (H3) a moderated mediation relationship between prior training, performance, fatigue, and sleep measured at the beginning of the competition phase, where the mediating effect of sleep on training to performance would be moderated by fatigue.

## 2. Materials and Methods

### 2.1. Participants

Twenty-four well-trained junior sprinters, competitive at the national level (top 8 in individual event) and with mean (±SD) age of 15.9 ± 1.6 years, height of 173 ± 9.1 cm, and weight of 65.0 ± 9.9 kg, volunteered to participate in this study. All participants were student athletes who had been competing for at least 2 years and trained 4 times per week minimum in the afternoon/evenings (between 3 pm and 7 pm) after school. The study protocol (ECP 87,16/17) was approved by the ethics committee at the university and carried out according to the Declaration of Helsinki. After comprehensive verbal and written explanation of the study, informed, written consent was signed by participants ≥18 years old, and parental consent and assent were obtained for participants <18 years old. Participants completed a health questionnaire so as to ensure they did not match Meeusen et al. [2] criteria of overtrained. All were free from allergies, endocrinological diseases (e.g., diabetes, thyroid disorders), and infectious diseases and were not taking prescribed medications throughout the study. No female athlete was menstruating on the days of sleep assessment, and females were not in the premenstrual phase (taken as 1 week prior to menstruation). Participants were asked to wear a sleep monitoring device for at least 5 nights inclusive of a night in the weekend. Four participants were excluded from subsequent analyses, because they had not worn the equipment as requested. The final sample size included in the analysis was *N* = 20 (*n* = 12 males and 8 females). Seven (*n* = 5 males, *n* = 2 females) athletes were suspected of maladaptation to the training cycle based on performance decrement and concomitantly high fatigue [2]. The remaining athletes who maintained or improved performance and fatigue (*n* = 13) were considered adapted.

### 2.2. Procedure

**Figure 1:**
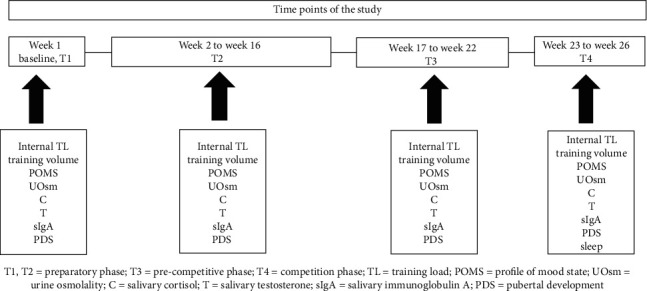
Schematic overview of the study design.

This study was a part of a larger observational study that elucidated training adaptation among track athletes throughout the sport training season. In the current study, sleep efficiency and quantity (taken as total sleep time) were measured using wrist actigraphy worn on the nondominant wrist. The actigraphy was worn during a week of habitual training, at the beginning of the competition phase for a minimum of 5 nights, inclusive of a night in the weekend. Participants' internal training loads (intensity and volume) were captured, within 15 to 30 minutes after each training session throughout the season [21]. The athletes' Profile of Mood State (POMS) [22], neuroendocrine response, immunological response, and spot urine osmolality (UOsm) were assessed on the first day of the preparatory phase/training cycle (baseline, T1) and then again midway within each mesocycle (periodization phase), namely, during (i) a 16-week preparatory phase (T2), (ii) a 6-week precompetitive phase (T3), and (iii) a 4-week competition phase (T4) over 26 weeks. Additionally, all participants completed the Pubertal Development Scale (PDS), a self-report measure of pubertal status [23]. The results indicated that participants were midpubertal to postpubertal. All measures were collected on a similar day (Monday) and at the same time (between 3 : 00 pm and 4 : 00 pm), particularly, to control for diurnal variations in the biological markers [24]. Athletes were asked to refrain from physical exercise within 24 h of the test sessions. In the hour before sample collection, athletes were asked (i) not to consume caffeine and food with high sugar content or acidity, (ii) not to eat a main meal, and (iii) not to brush or floss their teeth. Performance data were collected throughout the competitive period (T3 and T4). During the period of sample collection, participants were asked to maintain their habitual sleep pattern in an environment that was highly familiar to them. The week of sleep monitoring was free from official competition. Athletes followed the training prescribed by coaches throughout the mesocycles. An overview of the study protocol is presented in Figure 1.

### 2.3. Measures

#### 2.3.1. Training Monitoring

Training intensity and volume were captured using Foster et al.'s [21] session rating of perceived exertion (RPE) and the duration of the training session, respectively. The session RPE required participants to rate the intensity of all sessions using the category-ratio-10 Borg scale, multiplied by the duration of training sessions in minutes to yield internal loads. The session-RPE method was selected as it provides a simple and cost-effective indication of the varied physical demand imposed on sprint athletes with favorable comparison to heart rate and blood lactate measures of training loads [25]. Training load data were organized as the rolling average of 7-day (1-week) internal loads and as the rolling average of internal loads during a mesocycle (e.g., 6-week precompetitive phase). The average weekly load (acute) was divided by the average load across a mesocycle (chronic) to create the acute: chronic work ratio (ACWR) [26].

#### 2.3.2. Profile of Mood State

Fatigue and mood state were measured using the POMS questionnaire [22]. Participants were asked to complete 65 adjectives (e.g., lively, exhausted) loaded on 6 subscales (tension, depression, anger, vigor, fatigue, and confusion), describing how they were feeling in the “past week including today.” The 65 adjectives were anchored to a five-point intensity scale (not at all, a little, moderately, quite a bit, and extremely). The POMS provided a valid measure of overall mood as well as measures of six specific moods. The total mood disturbance (TMB) was computed by summing the five negative mood variables and subtracting the vigor score.

#### 2.3.3. Biological Assessments

Participants were required to rinse their mouth with water for approximately 1 minute. This was done 10 minutes prior to saliva sampling to remove any substance that may affect biological activity of the sample. Athletes provided timed 3-minute saliva samples by passive drooling into a preweighed sterile container. Samples were immediately transported to the laboratory on dry ice, where they were weighed and then centrifuged at 1500 × g for 15 minutes. The resulting supernatant was transferred to a 2 mL sterile tube and stored frozen at −80°C prior to analysis. The levels of cortisol, testosterone, and immunoglobulin A were assessed using commercially available enzyme-linked immunosorbent assay (ELISA) kits (Salimetrics, State College, USA). The analytical range of sensitivity for cortisol, testosterone, and immunoglobulin A was 0.33 nmol/L–82.77 nmol/L, 21.3 pmol/L–2080.5 pmol/L, and 2.5 *μ*g/mL–600 *μ*g/mL, respectively. Duplicate saliva samples were analyzed using 25 *μ*L of saliva per analysis of salivary cortisol and testosterone and 10 *μ*L per analysis of secretory immunoglobulin A. The mean intra-assay coefficient of variation was 5.8% for cortisol, 5.3% for testosterone, and 8.4% for immunoglobulin A. Salivary flow rate was determined by dividing the sample weight by sample timing, while secretory immunoglobulin A secretion rate was calculated as secretory immunoglobulin A concentration multiplied by flow rate.

### 2.4. Performance

Changes in individual performance were assessed during the competitive period using official competition times for each athlete's specialty event. Competition times were converted to scores using the International Association of Athletics Federation (IAAF) scoring table [27]. The score for the athlete's best race time during the precompetitive phase (PCP) was compared to the score for the best race time during the competition phase (CP) using the following equation:

(1) $$\begin{array}{c} \text{performance}=\frac{\text{CP}}{\text{CP}+\text{PCP}}, \end{array}$$

where PCP is the best performance score during the precompetitive phase and CP is the best performance score during the competition phase.

### 2.5. Sleep Monitoring

All participants were monitored continuously using commercially available wrist-worn accelerometers (ActiGraph wGT3X-BT, Pensacola, FL, USA) and daily self-reported sleep diaries. The epoch length of the accelerometer was set to 10 seconds. Athletes logged the times of going to bed and getting out of bed, times of lights off and lights on, number of times woken up during the night, and number and duration of naps taken during the days in sleep diaries. Participants were monitored for a week in their home environment and were instructed not to use accelerometers during showers or water-based activities. Mean behavioral activity over the entire recording period was calculated using ActiLife 6.0 software. Ten-second epochs were collapsed into 60-second epochs that have been the protocol for the study in adolescents [28]. Bedtime and get-up time were manually entered in the software to calculate sleep parameters. Wrist-worn accelerometers are noninvasive, cost-effective ways for analyzing sleep efficiency and quantity, with favorable comparison to the gold standard polysomnography [26]. Wrist-worn actigraphy was previously used to measure sleep in elite athletes [9, 29]. According to most studies, actigraphy has reasonable validity and reliability for the measurement of sleep in the healthy population [28]. However, its reliability can be improved when additional information is provided by a manually completed sleep log.

The participants' mean sleep behavior was analyzed from actigraph and sleep diaries over 5–7 days, for the following dependent variables:

1. Bedtime (hh : mm): self-reported time participant went to bed to try to sleep.
2. Get-up time (hh : mm): self-reported time participants got out of bed and stopped attempting to sleep.
3. Total time in bed (TIB, min): the amount of time spent in bed trying to sleep and nap.
4. Total sleep time (TST, min): sum of the actual time spent asleep overnight and during the day nap(s).
5. Sleep efficiency (SE %): total sleep time expressed as a percentage of the total time spent in bed trying to sleep (TST/TIB × 100%).
6. Sleep onset latency (SOL, min): the difference between time one went to bed and the first epoch of any sleep stage.
7. Wake after any sleep onset (WASO, min): the amount of wake time after the sleep onset.
8. Awakenings: the number of different awakening episodes as scored by the algorithm. This is sometimes referred to as frequency of awakenings (shown as the number of awakenings per night).

### 2.6. Statistical Analysis

Data were analyzed using Statistical Package for Social Sciences (SPSS, version 24, Chicago, IL, USA). The Shapiro–Wilk test was used to verify normality of dependent variables. All dependent variables were normally distributed except the biological variables (cortisol, C; testosterone, T; TC ratio; salivary immunoglobulin A, sIgA; salivary flow rate, sFR; sIgA secretion rate, sIgA SR). Log base 10 transformation achieved normality (Shapiro–Wilk test, *p* > 0.05) in these variables; hence, parametric tests were used for analyses. First, a “2-group: maladapted vs. adapted by 4-time: T1, T2, T3, T4” mixed model analysis of variance (ANOVA) was used to test for time effects and group by time interactions in weekly number of training days, training volume, internal loads, ACWR, POMS scales, and biological variables. To control for confounding factors, gender, pubertal development, and UOsm were included as covariates in the analyses of biological variables. Mauchly's test was conducted and Greenhouse–Geisser correction was applied if assumption of sphericity was violated. Significant main effect of timing was followed by Bonferroni post hoc test, to determine pairwise difference. Differences between groups at each time point were compared using independent *t*-test and reported as the mean difference and 95% confidence interval (CI). Second, Pearson correlation analysis was calculated for prior training (taken as mean of T1, T2, and T3 training data), performance, and the other variables that had significant group differences during T4 (at which time sleep parameters were collected). This was done to get an overview of their relationships. Finally, significant correlates were used to construct a moderated mediation model where sleep efficiency/quantity was a mediator between training and performance, and fatigue was the moderator. The other significant correlates were included in the moderated mediation model as covariates. The moderated mediation analysis was conducted using the PROCESS macro for SPSS version 3.4.2 model 14 [30]. The direct and the indirect effects were tested with bias-corrected nonparametric bootstrapping technique with 5000 bootstrap samples. To determine the moderated mediation effects, the mean center construction of product and a bias-corrected 95% CI were calculated for indices. When a 95% CI did not include zero, it indicated that the parameter was statistically significant. The effect sizes for ANOVA were analyzed using partial eta squared (*η*_*p*_^2^) to describe small (*η*_*p*_^2^ = 0.01), medium (*η*_*p*_^2^ = 0.06), and large (*η*_*p*_^2^ = 0.14) effects. The effect sizes for Bonferroni post hoc and independent *t*-tests were described using Cohen's *d* effect size (small: *d* ≤ 0.2, medium: 0.2 < *d* < 0.8, and large *d* ≥ 0.8) [31]. All tests that reported *p* value were set at the *p* < 0.05 level of significance.

## 3. Results

### 3.1. Characteristics of the Sample

Athletes suspected of maladaptation to training (*n* = 7) had ≥10% decrements in performance (see Table S1 in Supplementary Materials for performance data), as confirmed by official competitions and coaches. These athletes also have concomitantly high fatigue across the season relative to baseline (T1). The maladapted athletes had true and naturally occurring NFOR/OTS, as performance decline and concomitant high fatigue lasted for more than 3 weeks, and none had full performance recovery by the end of the athletic season (or by the end of this study carried out over the athletic season) [15]. Athletes considered adapted to the training program had <10% performance decrements or maintained/improved competition performance and fatigue (*n* = 13). The subsequent results are presented for athletes who were considered as adapted (AG) to the training program and those who were maladapted (MG). The descriptive characteristics of participants are shown in Table 1. Analyses of the participants' characteristics showed that there was no significant difference between groups (MG vs. AG, *p* > 0.05, Table 1).

### 3.2. Training Parameters

Changes in training parameters corresponding to the respective training phases are presented in Table 2. The result of examining all participants together demonstrated a significant change in weekly number of training days (*F* (3, 54) = 4.3, *p*=0.009, *η*_*p*_^2^ = 0.191) with no significant group effect and interaction. Post hoc Bonferroni test showed that the significant change was between T2 and T4 (*p*=0.033, *d* = 0.9). There was also significant change in weekly training volume (*F* (3, 54) = 18.5, *p* < 0.001, *η*_*p*_^2^ = 0.506) and internal training loads (*F* (3, 54) = 2.9, *p*=0.044, *η*_*p*_^2^ = 0.138), with no group effect and interaction. Weekly training volume was significantly lowered during T4 compared to the other training phases (*p* < 0.001, *d* range: 1.3–2.9). Relative to T1, internal training loads were increased during T2 (+33%, *p*=0.032, *d* = 0.9) and T3 (+30%, *p*=0.505, *d* = 0.5); then, they were decreased during T4 (−25%, *p*=0.634, *d* = 0.5) relative to T3. There was no significant change for ACWR during each of the respective training phases. Further, analysis of training parameters, using independent *t*-tests, revealed that there was no significant difference between groups during T1, T2, and T3 (Table 2). However, weekly training volume (*p*=0.028, *d* = 1.1) and internal training loads (*p*=0.032, *d* = 1.1) were significantly different between groups during T4. ACWR (*p*=0.072, *d* = 1.0) approached significance during T4.

### 3.3. POMS Pattern

Table 3 shows that all participants reported POMS fatigue scores less than 8 at baseline (T1: 6.0 ± 1.9), confirming that they were not already in a fatigue state at the beginning of the study [15]. Fatigue significantly increased at all phases of the season relative to baseline (*F* (3, 54) = 7.7, *p* < 0.001, *η*_*p*_^2^ = 0.299). There were significant group effects (*F* (1, 18) = 35.7, *p* < 0.001, *η*_*p*_^2^ = 0.665) and interactions (*F* (3, 54) = 7.8, *p* < 0.001, *η*_*p*_^2^ = 0.302) for fatigue. During T2, fatigue (9.7 ± 5.7) was significantly (*p*=0.005, *d* = 0.9) increased relative to T1. Fatigue during T3 (8.2 ± 5.7) remained similar to that during T2 but significantly increased relative to baseline (*p*=0.001, *d* = 0.5). Fatigue during T4 (7.8 ± 5.0) was also similar to that during T2 and T3 but significantly increased relative to T1 (*p*=0.004, *d* = 0.5). Independent *t*-tests showed no significant group difference during T1 but significantly higher fatigue in MG relative to AG athletes during T2 (*p*=0.034, *d* = 1.0), T3 (*p* < 0.001, *d* = 2.7), and T4 (*p* < 0.001, *d* = 3.6). There was no significant time effect and interaction for TMD and the other POMS subscales (tension, anger, vigor, depression, and confusion). There was no group effect for anger, vigor, and confusion; however, significant group effects were seen for TMD (*F* (1, 18) = 7.6, *p*=0.013, *η*_*p*_^2^ = 0.296) and tension (*F* (1, 18) = 6.3, *p*=0.022, *η*_*p*_^2^ = 0.259), and depression approached significance (*F* (1, 18) = 4.2, *p*=0.056, *η*_*p*_^2^ = 0.188). The MG displayed greater depression (*p*=0.029, *d* = 1.1) and tension (*p*=0.027, *d* = 1.2) during T4 (Table 3).

### 3.4. Biological Response

For the biological markers, there was no significant time effect, group effect, and interaction after controlling for gender, UOsm, and pubertal development (see Table S2 in Supplementary Materials for biochemical response). Independent *t*-test showed significantly greater levels of absolute sIgA (*p*=0.006, *d* = 1.3) in the AG relative to MG during T4.

### 3.5. Sleep Assessment

Raw values of sleep parameters, obtained from sleep actigraphy and sleep diary at the start of T4, are presented in Table 4 for both groups of athletes. The results (mean difference (95% CI)) show that total sleep time (−51.4 (−71.9, −32.1)), sleep efficiency (−13.6 (−18.2, −9.0), sleep onset latency (7.4 (1.4, 13.4)), and wake after sleep onset (16.3 (2.3, 30.3)) were significantly different in adapted athletes compared to maladapted athletes. There was no significant difference for time in bed (16.9 (−20.0, 53.8)), bedtime (0 : 32 (−0:21, 1 : 26)), get-up time (0 : 49 (−0 : 13, 1 : 52)), and number of awakenings (14.5 (−11.9, 40.8)) between both groups of athletes.

### 3.6. Preliminary Analyses of Relationships between Training, Sleep, Fatigue, and Performance

Pearson correlations were calculated on pooled data for both MG and AG using prior training (mean of T1, T2, and T3 training data), performance (relative change across competitive period: T3, T4), fatigue (during T4), and sleep parameters that had significant between-group differences during T4.  Table 5 shows that performance correlated negatively with training volume (*p* < 0.001), fatigue (*p*=0.001), and total mood disturbance (*p*=0.055). Performance correlated positively with absolute sIgA (*p*=0.008), total sleep time (*p*=0.005), and sleep efficiency (*p* < 0.001). In addition, training volume correlated positively with fatigue (*p*=0.001) but negatively with absolute IgA (*p*=0.024), total sleep time (*p*=0.001), sleep efficiency (*p* < 0.001), and wake after sleep onset (*p*=0.011). Fatigue correlated positively with depression (*p* < 0.001) and tension (*p*=0.004) but negatively with absolute sIgA (*p*=0.045), total sleep time (*p* < 0.001), and sleep efficiency (*p* < 0.001). Finally, total sleep time significantly correlated negatively with tension (*p*=0.021), depression (*p*=0.01), and TMS (*p*=0.001). Similarly, sleep efficiency correlated negatively with depression (*p*=0.044) and TMS (*p*=0.026) but not with tension (*p*=0.348). Both total sleep time (*p*=0.016) and sleep efficiency (*p*=0.008) correlated positively with absolute sIgA.

### 3.7. Moderated Mediation Analysis

Moderated mediation analyses were conducted to identify the relationships between prior training, performance, fatigue, and sleep efficiency/quantity at T4 (the only point where sleep was evaluated). Prior to framing the moderated mediation model, gender, age, absolute sIgA, tension, and depression were tested to see if they had a significant relation with any of the variables. Gender and age were not significantly related to any of the variables and thus were not included further in the analyses. However, since absolute sIgA was significantly related to performance, sleep efficiency, total sleep time (quantity), and fatigue, it was included in the model as covariate. Tension and depression were significantly related to the sleep parameters and thus were also treated as covariates (Table 5).

**Figure 2:**
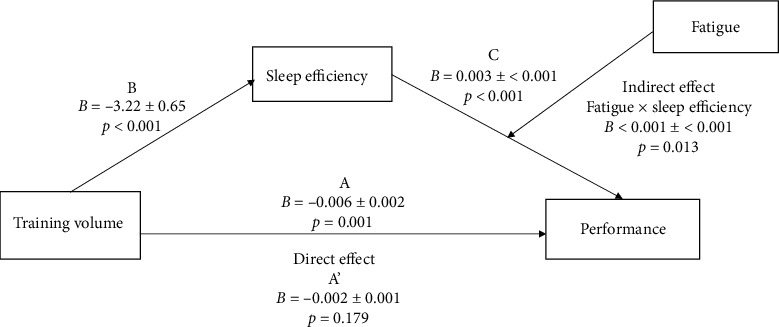
Conceptual model of prior training volume to performance relationship illustrating moderated mediation effects of sleep efficiency x fatigue (*B* = unstandardized coefficients ± standard errors).

To frame the moderated mediation models, the sleep parameters of efficiency and total sleep time collected during the first week of T4 were treated as mediators between prior training volume and performance relationship. Fatigue was treated as a moderator between the sleep efficiency/total sleep time and performance relationship. The first regression analysis showed that training volume had significant negative effects on performance (*β* = −0.68, *t* = −4.13, *p*=0.001; Figure 2, Path A). The second regression analysis showed a negative and significant effect of prior training volume on sleep efficiency (*β* = −0.76, *t* = −4.94, *p* < 0.001; Figure 2, Path B) and of prior training volume on total sleep time (*β* = −0.71, *t* = −4.23, *p* < 0.001) as well as a positive significant effect of sleep efficiency on performance (*β* = 1.40, *t* = 5.23, *p* < 0.001; Figure 2, Path C). The effect of total sleep time on performance (*β* = 0.19, *t* = −1.09, *p*=0.296) was not significant. The indirect effect of sleep efficiency on performance (*β* = −0.24, *t* = −2.27, *p*=0.013) was significant, but the indirect effect of total sleep time on performance (*β* = < 0.001, *t* = 0.99, *p*=0.339) was not significant. Concerning the covariates, neither absolute sIgA, tension, nor depression was significant in the model. These findings indicated that the mediating effect of sleep efficiency on prior training volume to performance relationships was moderated by fatigue. Total sleep time was not a significant mediator between prior training volume and performance relationship. Overall, the predictors in the model explained 91% of the variance observed in performance (*F* (6, 13) = = 22.60, *p* < 0.001). The inclusion of the interaction between sleep efficiency and fatigue led to a change of *R*^2^ = 0.06 (*F* (1, 13) = 8.25, *p*=0.013), whereas the inclusion of the interaction between total sleep time and fatigue led to a change of *R*^2^ = 0.01 (*F* (1, 13) = 0.99, *p*=0.339).

**Figure 3:**
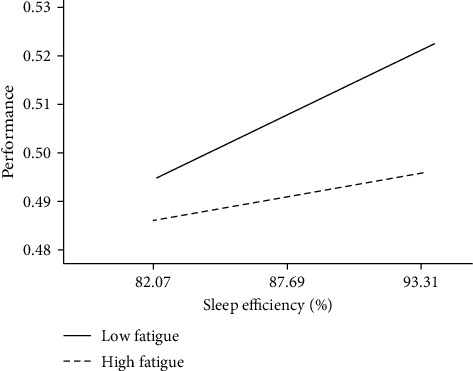
Moderating effect of fatigue on the association between performance and sleep efficiency.

To visually describe the moderation effect, the effect of sleep efficiency on performance was calculated for low (1SD below the mean) and high (1SD above the mean) levels of the moderator, fatigue (Figure 3). The simple slope test revealed that, for athletes with low fatigue, higher sleep efficiency was strongly related to better performance *B* = 0.003 ± <0.001, *t* = 5.50, *p*=0.001, 95% CI (0.001, 0.004). For participants with high fatigue levels, the indirect effect became less significant, *B* = 0.001 ± <0.001, *t* = 2.91, *p*=0.012, 95% CI (<0.001, 0.002), indicating that prior training volume has indirect effect on performance through sleep efficiency which is contingent on fatigue, such that higher levels of fatigue decrease the indirect effect's magnitude. Overall, the bias-corrected percentile bootstrap method further revealed a significant moderated mediation effect, *B* = 0.002 ± 0.002, 95% CI (<0.001, 0.007), in which fatigue at T4 moderated the mediating effect of sleep efficiency by buffering its influence on performance results.

## 4. Discussion

The paper adds to the existing literature on sleep, overtraining, and athletes. Uniquely, the study examined sleep in the understudied elite adolescent athletic (sprint) population: (i) by comparing sleep efficiency/quantity at the start of competition phase (T4) in adolescent sprinters who adapted to a prior training cycle relative to those who maladapt and (ii) by examining the influence of prior training, sleep, and fatigue on performance through a moderated mediation model. Here, we found significant differences in sleep efficiency/quantity at the start of T4 in athletes who adapted to a prior training cycle (22 weeks of preparatory and precompetitive training) in comparison to those who maladapted (NFOR/OTS affected athletes). In addition, through a cross-sectional analysis, we found that impaired sleep through greater prior training volume was negatively related to performance through fatigue.

It is important to note that increased fatigue and declined performance are hallmarks in the overtraining response continuum and can be used to determine whether a training cycle is maladapted after it has concluded [2, 4]. In the current study, adolescent sprinters who demonstrated ≥10% decline in performance and had concomitantly greater levels of fatigue after 22 weeks of preparatory and precompetitive training showed significantly lower sleep efficiency (82 ± 3%) and quantity (measured as total sleep time, 394 ± 20 min) than athletes who maintained or improved performance and fatigue (91 ± 3% and 425 ± 33 min). Other sleep parameters such as sleep latency and wake after sleep onset were also better (lower) in athletes who adapted to the training cycle. Bedtime, get-up time, and time in bed were, however, not significantly different between both groups of athletes, suggesting sleep disturbance in athletes who developed maladaptation possibly as a result of the prior training. As this is the first study to examine sleep in a group of solely adolescent sprinters (age: 14–19 years) who developed training maladaptation versus those who adapt, comparisons were difficult to make. Our results paralleled findings from other studies that examined sleep in overreached versus nonoverreached senior triathletes [8], female swimmers [5], and high school mixed with university swimmers (100–200 m) [6]. However, caution must be taken with these comparisons as sleep patterns in the female population and adolescents can be completely different from the male population and adults, respectively. This is because sleep patterns are affected by the mensural cycle and hormonal changes [32] and by the biological delay in timing of sleep onset in adolescents, causing them to stay awake later [17, 20].

In the current study, sleep efficiency (88 ± 6%) obtained from the pooled data for adapted and maladapted sprint athletes was considered good based on the <85% criteria used to define sleep disturbance [33]. Sleep quantity (6.91 ± 0.53 h) on the other hand was less than the 8.5 to 9.5 h sleep requirement in adolescents [34]. Sleep efficiency and quantity in the current study were, however, greater than values reported by Leeder and colleagues [29] in a cohort of elite athletes (81 ± 6% and 6.55 ± 0.43 h) under normal training conditions. Sleep efficiency in our maladapted sprint athletes (82 ± 3%) is considered poor (<85%) based on cutoff for sleep disturbance [33]. Sleep efficiency in maladapted athletes was similar to values reported in Wall and colleagues' [6] study on overreached high school mixed with university sprint swimmers (82 ± 4%), but significantly lower than values reported by Hausswirth and others' [8] study on FOR senior triathletes (88 ± 2%). Again, these differences could be based on the fact that adolescents have different wake after sleep onset, total sleep time, and sleep efficiency compared with adults [17] and could also be dependent on the equipment used [26].

Previous studies are limited in that they did not take into consideration the interrelations between training, sleep, fatigue, and performance [5, 6, 8]. Therefore, our study offered a new contribution which may help to elucidate whether sleep disturbance is an etiological mechanism of FOR/OTS or simply just a symptom. Contrary to the existing literature, there was no association between internal loads and performance [35] or association between internal loads and sleep efficiency/quantity [10, 19]. However, greater prior training volume was negatively associated with performance, which is consistent with previous studies [5]. Moreover, prior training volume was negatively related to performance through sleep efficiency, indicating that greater training volume was related to sleep disturbance which in turn was related to decreased performance. These results contradict the theory that sleep is proportional to restorative needs, meaning the more one trains, the more one would want to sleep [36], but are consistent with findings of impaired sleep during periods of hard training or when athletes are overreached [6, 8]. Accumulating evidence which demonstrated that excessive training causes suppressed immune response [9], restlessness during sleep, heavy legs during sleep, and soreness/pain as a result of muscle damage may partially explain impaired sleep after excessive training [1]. Additionally, we found that greater sleep efficiency was related to better performance, in line with previous studies that consistently demonstrated that greater (better) sleep efficiency is related to success in competition [9]. For example, Mah and colleagues [14] demonstrated that a 2 h sleep extension for a period of 5 to 7 weeks caused significant improvement in sprint test time as well as fatigue, vigor, and performance during practice and competition in colligate basketball players.

Finally, our results showed that fatigue moderated the relationship between sleep efficiency and performance, meaning fatigue moderated the indirect effect of prior training volume on performance through sleep efficiency. Specifically, the effect of sleep efficiency on performance was stronger when fatigue score was low than when fatigue score was high. These findings are consistent with previous research which found that sleep disturbance may potentiate fatigue [19]. Accordingly, faced with a low sleep efficiency induced by high training volume, athletes with high levels of fatigue were more likely to experience performance decrements than athletes with low levels of fatigue. This is because individuals experiencing high levels of fatigue typically experience lack of energy, muscle pain, feelings of lassitude, decreased feelings of motivation and alertness, and changes in perception and mood as compared to individuals with low levels of fatigue [37]. These may have occurred through the roles of neurotransmitters 5-hydroxytryptamine (5HT, serotonin) and dopamine which play some key roles in tiredness and sleep [7]. Sleep deprivation causes a significant increase in 5HT and a significant decrease in dopamine binding potential in certain brain areas [38]. This means that a low ratio of 5HT to dopamine favors increased arousal, motivation, and optimal neuromuscular coordination, being thus beneficial to performance. A high ratio of 5HT to dopamine, on the other hand, favors decreased motivation, lethargy, tiredness, and loss of motor coordination and thus may negatively affect performance [39]. These may explain the relationship between sleep, fatigue, and performance in potentiating overreaching/overtraining; however, these were not measured in the present study.

This study presents several strengths. This is one of the few studies to assess sleep in adolescent athletes, in particular, sprint athletes who were maladapted (NFOR/OTS) to a training cycle. To our knowledge, our study was the first to examine the relationship between prior training volume, sleep, fatigue, and performance among elite sprint adolescents so as to elucidate the pathways linking sleep to NFOR/OTS. Based on our findings, we recommend the following: (i) effort should be made to enhance sleep efficiency during periods of high training volume to prevent maladaptation (high fatigue and low performance) to training. (ii) Practitioners should recognize that prior training volume may have a more profound impact on performance through sleep efficiency and fatigue than internal loads (perceived exertion x volume) itself. (iii) The significantly greater wake after sleep onset in maladapted athletes relative to adapted athletes during the start of the competition phase, in conjunction with the nonsignificant effect of total sleep time but the significant effect for sleep efficiency, to mediate the training to performance relationship, means that practitioner should not solely focus on extending sleep time, but optimize conditions that promote continuous and quality sleep in athletes.

Nevertheless, some limitations of the study should be underscored. First, our findings may not be generalizable to other subpopulations, because of the sample size. Such further testing of these hypotheses with larger populations is needed, so as to cement the mechanism linking sleep, fatigue, and performance to NFOR/OTS. It is also recommended that future studies compare sleep during competition phase to sleep at baseline. This would illuminate whether sleep deterioration over the season is related to maladaptation. Second, although measuring sleep in a field setting provides a mirror image of the ecological sleep-wake behavior, it may result in inaccuracies due to small losses in measurement sensitivity associated with equipment. Accuracy in actual sleep-wake behavior may be lost since actigraphy is reliant on movement to indicate wakefulness and lack of movement to indicate sleep [28]. Hence, periods of wakefulness not accompanied by movement could be scored as sleep. To reduce this scoring error, sleep diaries were used to augment measurements obtained from actigraphy. Finally, in our study, we used concomitant increase in fatigue, mood disturbance, and performance decrements to diagnose athletes as adapted to training or maladapted to training. This method of diagnosis should be approached with caution as FOR/NFOR/OTS is difficult to diagnose and requires a battery of biomarkers relating to psychological, physiological, biological, and immunological characteristics [2, 3, 15]. These approaches can be impractical for field studies since they can be invasive. Thus, end of a training cycle, levels of fatigue, mood state, and performance decrement which have successfully been used to diagnose athletes as overtrained [2, 4] were used as the more practical alternatives.

## 5. Conclusions

Athletes who had progressive increase in fatigue and performance decrements across a training cycle (maladapted athletes) experienced less efficient sleep at the start of the competition phase relative to athletes who maintained or improved their fatigue and performance (adapted athletes). Moreover, greater prior training volumes were related to performance decrements through sleep efficiency and fatigue, meaning that impaired sleep as a result of high prior training volume may induce fatigue and lead to a reduction in performance. These findings suggest that special attention should be paid to athletes' sleep efficiency during high volume training.

## Figures and Tables

**Table 1:** Descriptive characteristics of participants disaggregated based on athletes considered as adapted to and maladapted to training. Data are presented as mean ± SD.

Variable	Overall (*N* = 20)	MG (*n* = 7)	AG (*n* = 13)	*p* value	*d*
Age (year)	15.9 ± 1.7	16.6 ± 1.9	15.5 ± 1.5	0.160	0.6
Height (cm)	173.0 ± 9.9	173.7 ± 10.4	172.6 ± 10.0	0.814	0.1
Weight (kg)	65.2 ± 12.7	67.1 ± 14.6	64.17 ± 12.17	0.634	0.2
BMI (kg/m^2^)	21.6 ± 2.3	22.0 ± 2.8	21.3 ± 2.1	0.548	0.3
%BF	13.0 ± 5.0	12.0 ± 3.9	13.6 ± 5.6	0.510	0.4

MG: maladapted group; AG: adapted group; *d*: Cohen' *d* effect size; %BF: percent body fat.

**Table 2:** Mean ± SD and between-group effects as mean difference (95% confidence interval) for training parameters of participants according to training phases.

Variables	T1	T2	T3	T4	Total between-group effects, MG-AG (95% CI)
Weekly no. TD	5.3 ± 0.7	5.4 ± 0.6	5.1 ± 0.4	4.8 ± 0.7^b^	
MG	5.1 ± 0.7	5.4 ± 0.5	5.0 ± 0.4	5.0 ± 0.0	−0.03 (−0.3, 0.5)†
AG	5.4 ± 0.7	5.4 ± 0.6	5.2 ± 0.4	4.5 ± 0.9	

Weekly TV	12.2 ± 1.8	12.1 ± 1.8	10.5 ± 4.0	6.7 ± 1.9^abc^	
MG	12.0 ± 1.9	12.5 ± 1.3	11.2 ± 3.9	8.5 ± 7.7‡	1.1 (−0.3, 2.4)†
AG	12.3 ± 1.8	11.9 ± 2.0	10.1 ± 4.2	5.7 ± 1.1	

Internal TL	3003.7 ± 764.9	3995.0 ± 1443.0^a^	3928.8 ± 2332.6^a^	2960.0 ± 1471.9	
MG	2739.1 ± 635.1	3997.1 ± 472.8	4165.0 ± 2869.7	3897.9 ± 1500.3‡	350.7 (−706.8, 1408.1)†
AG	3146.8 ± 813.53	3993.8 ± 1784.7	3801.5 ± 2108.9	2455.0 ± 1231.0	

ACWR	1.0 ± 0.3	1.0 ± 0.2	1.1 ± 0.3	0.9 ± 0.2	
MG	0.9 ± 0.2	1.0 ± 0.2	1.0 ± 0.3	1.0 ± 0.2	0.01 (−0.01, 0.03)
AG	1.0 ± 0.3	1.1 ± 0.2	1.1 ± 0.3	0.8 ± 0.2	

^†^Significant time effect (repeated measure ANOVA, *p* < 0.05). ^‡^Significant group difference (independent *t*-test, *p* < 0.05). ^a^Significantly different from T1. ^b^Significantly different from T2. ^c^Significantly different from T3. No. TD: number of training days; TV: training volume in hours; TL: training load in arbitrary unit; ACWR: acute chronic work ratio; MG: maladapted group; AG: adapted group.

**Table 3:** Mean ± SD and between-group effects as mean difference (95% confidence interval) for fatigue, total mood state, and other POMS scores corresponding to different phases of an athletic season.

Variable	T1	T2	T3	T4	Total between-group effects, MG-AG (95% CI)
Fatigue	6.0 ± 1.9	9.7 ± 5.7^a^	8.2 ± 5.7^a^	7.8 ± 5.0^a^	
MG	6.9 ± 1.1	13.3 ± 5.7	14.4 ± 4.0^‡^	13.7 ± 2.9^‡^	6.4 (3.9, 8.9)^*∗*†^
AG	5.5 ± 2.1	7.8 ± 4.8	4.9 ± 3.0	4.5 ± 2.1	

Tension	8.4 ± 4.7	8.0 ± 5.0	7.4 ± 4.8	7.2 ± 4.1	
MG	11.1 ± 3.6	10.6 ± 4.4	10.1 ± 4.7	9.9 ± 3.6^‡^	4.2 (0.7, 7.7)
AG	6.9 ± 4.6	6.5 ± 4.9	5.9 ± 4.2	5.7 ± 3.7	

Anger	6.6 ± 5.8	6.1 ± 5.7	6.0 ± 5.4	5.6 ± 4.4	
MG	7.1 ± 6.8	9.6 ± 5.4	7.4 ± 5.2	7.7 ± 4.9	3.1 (-1.4, 7.7)
AG	6.0 ± 5.3	4.2 ± 5.0	5.2 ± 5.6	4.5 ± 3.9	

Vigor	14.6 ± 3.4	13.7 ± 2.9	13.8 ± 3.5	13.5 ± 3.6	
MG	12.6 ± 2.7	13.0 ± 2.8	13.4 ± 2.5	12.6 ± 2.6	-1.1 (-3.9, 1.8)
AG	14.0 ± 2.9	14.0 ± 2.9	13.9 ± 4.0	13.9 ± 4.0	

Depression	7.4 ± 4.3	6.6 ± 5.8	4.8 ± 4.7	5.1 ± 4.0	
MG	8.6 ± 4.9	10.0 ± 5.4^‡^	6.4 ± 5.3	7.7 ± 3.7^‡^	3.4 (-0.1, 7.0)
AG	6.8 ± 4.1	4.7 ± 5.3	3.9 ± 4.3	3.7 ± 3.6	

Confusion	5.7 ± 3.9	5.7 ± 4.4	5.5 ± 3.3	5.6 ± 3.1	
MG	7.0 ± 4.9	6.7 ± 5.4	6.7 ± 3.5	7.0 ± 3.3	2.0 (-1.1, 5.1)
AG	4.9 ± 3.2	5.0 ± 3.8	4.9 ± 3.2	4.9 ± 2.8	

TMD	19.4 ± 17.7	22.3 ± 24.2	18.0 ± 19.7	17.8 ± 18.1	
MG	27.8 ± 16.7	37.3 ± 21.7^‡^	31.7 ± 20.7^‡^	33.4 ± 17.6^‡^	20.3 (4.8, 35.9)
AG	14.9 ± 17.0	14.2 ± 22.1	9.3 ± 12.0	9.3 ± 12.0	

^†^Significant time effect (repeated measure ANOVA, *p* < 0.05). ^∗^Significant group × time interaction (repeated measure ANOVA, *p* < 0.05). ‡Significant group difference (independent *t*-test, *p* < 0.05). ^a^Significantly different from T1. MG: maladapted group; AG: adapted group; TMD: total mood disturbance.

**Table 4:** Mean ± SD of sleep actigraphy data during the competitive phase in two groups of athletes considered as adapted and maladapted to training.

Variables	All (*N* = 20)	MG (*n* = 7)	AG (*n* = 13)	*p* value	*d*
Bedtime (hh:mm)	10 : 52 ± 0 : 55	11 : 13 ± 1 : 00	10 : 41 ± 0 : 52	0.226	0.4
Get-up time (hh:mm)	6 : 45 ± 1 : 06	7 : 17 ± 1 : 19	6 : 28 ± 0 : 54	0.119	0.7
TIB (min)	473.7 ± 37.4	484.6 ± 36.4	467.8 ± 38.0	0.349	0.5
TST (min)	414.5 ± 32.1	394.3 ± 20.4	425.3 ± 32.6	**0.036^*∗*^**	1.1
SE%	87.7 ± 5.5	81.5 ± 2.9	91.0 ± 3.4	**<0.001^*∗*^**	3.0
SOL (min)	9.9 ± 7.0	14.8 ± 7.5	7.3 ± 5.3	**0.018^*∗*^**	1.2
WASO (min)	38.8 ± 15.9	49.4 ± 16.8	33.1 ± 12.7	**0.025^*∗*^**	1.1
No. of awakenings	23.6 ± 27.0	33.0 ± 32.6	18.5 ± 23.3	0.263	0.5

MG: maladapted group; AG: adapted group; *d*: Cohen' *d* effect size; TIB: time in bed; TST: total sleep time; SE%: sleep efficiency; SOL: sleep onset latency; WASO: wake after sleep onset.

**Table 5:** Correlation calculated on pooled data for both adapted and maladapted athletes.

	1	2	3	4	5	6	7	8	9	10	11	12
(1) Performance	—	—	—	—	—	—	—	—	—	—	—	—
(2) Internal TL	−0.27	—	—	—	—	—	—	—	—	—	—	—
(3) Training volume	−0.79^*∗∗*^	0.14	—	—	—	—	—	—	—	—	—	—
(4) Fatigue	−0.69^*∗∗*^	0.57^*∗∗*^	0.69^*∗∗*^	—	—	—	—	—	—	—	—	—
(5) Tension	−0.16	0.47^*∗*^	0.21	0.62^*∗∗*^	—	—	—	—	—	—	—	—
(6) Depression	−0.35	0.45^*∗*^	0.34	0.72^*∗∗*^	0.80^*∗∗*^	—	—	—	—	—	—	—
(7) Absolute sIgA	0.55^∗^	−0.01	−0.50^*∗*^	−0.45^*∗*^	−0.22	0.35	—	—	—	—	—	—
(8) TMD	−0.44	0.61^*∗∗*^	0.44	0.84^*∗∗*^	0.85^*∗∗*^	0.91^*∗∗*^	−0.33	—	—	—	—	—
(9) TST	0.60^*∗∗*^	−0.25	−0.70^*∗∗*^	−0.83^*∗∗*^	−0.51^∗^	−0.56^*∗∗*^	0.53^*∗*^	−0.69^*∗∗*^	—	—	—	—
(10) SE%	0.91^*∗∗*^	−0.29	−0.76^*∗∗*^	−0.76^*∗∗*^	−0.22	−0.46^*∗*^	0.58^*∗∗*^	−0.50^*∗∗*^	−0.62^*∗∗*^	—	—	—
(11) SOL	−0.48^*∗*^	0.35	0.23	0.42	0.20	0.13	0.22	0.35	−0.52^*∗*^	−0.43	—	—
(12) WASO	−0.66	0.03	0.56^∗^	0.17	−0.07	−0.19	−0.29	0.08	−0.19	−0.55^*∗*^	0.29	—

^*∗*^ *p* < 0.05; ^*∗∗*^*p* < 0.001. TL: training load; sIgA: secretory immunoglobulin A; TMD: total mood disturbance; TST: total sleep time; SE%: sleep efficiency; SOL: sleep onset latency; WASO: wake after sleep onset.

## Data Availability

The data used to support the findings of this study are available from the corresponding author upon request.

## Supplementary Materials

Supplementary Materials Table S1: performance data for each sprinter across the competitive phase of the athletic season. Table S2: the biochemical response of sprinters corresponding to different phases of the athletic season.

## References

[1] Lastella M., Vincent G. E., Duffield R. (2018). Can sleep be used as an indicator of overreaching and overtraining in athletes?. *Frontiers in Physiology*.

[2] Meeusen R., Duclos M., Foster C (2013). Prevention, diagnosis, and treatment of the overtraining syndrome: joint consensus statement of the European college of sport science and the American college of sports medicine. *Medicine and Science in Sports and Exercise*.

[3] Cadegiani F. A., Kater C. E. (2017). Hormonal aspects of overtraining syndrome: a systematic review. *BMC Sports Science, Medicine and Rehabilitation*.

[4] Kenttä G., Hassmén P. (1998). Overtraining and recovery. *Sports Medicine*.

[5] Taylor S. R., Rogers G. G., Driver H. S. (1997). Effects of training volume on sleep, psychological, and selected physiological profiles of elite female swimmers. *Medicine & Science in Sports & Exercise*.

[6] Wall S. P., Mattacola C. G., Swanik C. B., Levenstein S. (2003). Sleep efficiency and overreaching in swimmers. *Journal of Sport Rehabilitation*.

[7] Budgett R. (1998). Fatigue and underperformance in athletes: the overtraining syndrome. *British Journal of Sports Medicine*.

[8] Hausswirth C., Louis J., Aubry A., Bonnet G., Duffield R. O. B., Le Meur Y. (2014). Evidence of disturbed sleep and increased illness in overreached endurance athletes. *Medicine & Science in Sports & Exercise*.

[9] Walsh N. P., Halson S. L., Sargent C. (2020). Sleep and the athlete: narrative review and 2021 expert consensus recommendations. *British Journal of Sports Medicine*.

[10] Vitale K. C., Owens R., Hopkins S. R., Malhotra A. (2019). Sleep hygiene for optimizing recovery in athletes: review and recommendations. *International Journal of Sports Medicine*.

[11] Reilly T., Waterhouse J. (2009). Sports performance: is there evidence that the body clock plays a role?. *European Journal of Applied Physiology*.

[12] Mah C. D., Mah K. E., Kezirian E. J., Dement W. C. (2011). The effects of sleep extension on the athletic performance of collegiate basketball players. *Sleep*.

[13] Van Dongen P., Baynard M. D., Maislin G., Dinges D. F. (2004). Systematic interindividual differences in neurobehavioral impairment from sleep loss: evidence of trait-like differential vulnerability. *Sleep*.

[14] Hirotsu C., Tufik S., Andersen M. L. (2015). Interactions between sleep, stress, and metabolism: from physiological to pathological conditions. *Sleep Science*.

[15] Cadegiani F. A., da Silva P. H. L., Abrao T. C. P., Kater C. E. (2020). Diagnosis of overtraining syndrome: results of the endocrine and metabolic responses on overtraining syndrome study: eros-diagnosis. *Journal of Sports Medicine*.

[16] Roberts S. S. H., Teo W.-P., Warmington S. A. (2019). Effects of training and competition on the sleep of elite athletes: a systematic review and meta-analysis. *British Journal of Sports Medicine*.

[17] Taylor L., Chrismas B. C. R., Dascombe B., Chamari K., Fowler P. M. (2016). The importance of monitoring sleep within adolescent athletes: athletic, academic, and health considerations. *Frontiers in Physiology*.

[18] Nedelec M., Aloulou A., Duforez F., Meyer T., Dupont G. (2018). The variability of sleep among elite athletes. *Sports Medicine-Open*.

[19] Watson A., Brickson S. (2018). Impaired sleep mediates the negative effects of training load on subjective well-being in female youth athletes. *Sports Health: A Multidisciplinary Approach*.

[20] Crowley S. J., Carskadon M. A. (2010). Modifications to weekend recovery sleep delay circadian phase in older adolescents. *Chronobiology International*.

[21] Foster C., Florhaug J. A., Franklin J. (2001). A new approach to monitoring exercise training. *The Journal of Strength and Conditioning Research*.

[22] McNair D. M., Lorr M., Droppleman L. F. (1981). *Manual for the Profile of Mood States*.

[23] Petersen A. C., Crockett L., Richards M., Boxer A. (1988). A self-report measure of pubertal status: reliability, validity, and initial norms. *Journal of Youth and Adolescence*.

[24] Kobayashi H., Song C., Ikei H., Park B.-J., Kagawa T., Miyazaki Y. (2017). Diurnal changes in distribution characteristics of salivary cortisol and immunoglobulin A concentrations. *International Journal of Environmental Research and Public Health*.

[25] Haddad M., Stylianides G., Djaoui L., Dellal A., Chamari K. (2017). Session-RPE method for training load monitoring: validity, ecological usefulness, and influencing factors. *Frontiers in Neuroscience*.

[26] Quante M., Kaplan E. R., Cailler M. (2018). Actigraphy-based sleep estimation in adolescents and adults: a comparison with polysomnography using two scoring algorithms. *Nature and Science of Sleep*.

[27] Spiriev B., Spiriev A. (2014). *IAAF Scoring Tables of Athletics*.

[28] Sadeh A. (2011). The role and validity of actigraphy in sleep medicine: an update. *Sleep Medicine Reviews*.

[29] Leeder J., Glaister M., Pizzoferro K., Dawson J., Pedlar C. (2012). Sleep duration and quality in elite athletes measured using wristwatch actigraphy. *Journal of Sports Sciences*.

[30] Hayes A. F. (2017). Introduction to mediation, moderation, and conditional process analysis. *A Regression-Based Approach*.

[31] Cohen J. (1988). *Statistical Power Analysis for the Behavioral Sciences*.

[32] Baker F. C., Lee K. A. (2018). Menstrual cycle effects on sleep. *Sleep Medicine Clinics*.

[33] Lacks P., Morin C. M. (1992). Recent advances in the assessment and treatment of insomnia. *Journal of Consulting and Clinical Psychology*.

[34] Williams J. A., Zimmerman F. J., Bell J. F. (2013 Jan). Norms and trends of sleep time among US children and adolescents. *JAMA Pediatrics*.

[35] Balsalobre-Fernández C., Tejero-González C. M., del Campo-Vecino J. (2014). Relationships between training load, salivary cortisol responses and performance during season training in middle and long distance runners. *PLoS One*.

[36] Adam K. (1980). Sleep as a restorative process and a theory to explain why. *Progress in Brain Research*.

[37] Schiphof-Godart L., Roelands B., Hettinga F. J. (2018). Drive in Sports: how mental fatigue affects endurance performance. *Frontiers in Psychology*.

[38] Davis J. M., Alderson N. L., Welsh R. S. (2000). Serotonin and central nervous system fatigue: nutritional considerations. *The American Journal of Clinical Nutrition*.

[39] Volkow N. D., Tomasi D., Wang G.-J. (2012). Evidence that sleep deprivation downregulates dopamine D2R in ventral striatum in the human brain. *Journal of Neuroscience*.

