# Supplementary material for: Evidence That Sleep Is an Indicator of Overtraining during the Competition Phase of Adolescent Sprinters
Source: J Sports Med (Hindawi Publ Corp). 2021 Apr 3;2021:6694547. doi: 10.1155/2021/6694547 (PMC8041504; doi:10.1155/2021/6694547)
Supplement: Supplementary Materials — Table S1: performance data for each sprinter across the competitive phase of the athletic season. Table S2: the biochemical response of sprinters corresponding to different phases of the athletic season. [file 6694547.f1.zip › 6694547.f1/Supplementaeary Table 2 (1).docx]

Table S2: Mean ±SD and between group effects as mean difference (95% confidence interval) for salivary markers corresponding to training phases.

| Variable | T1 | T2 | T3 | T4 | Total between group effects  MG-AG (95% CI) |
| --- | --- | --- | --- | --- | --- |
| **C** | 2.42 ± 1.96 | 4.08 ± 4.67 | 5.16 ± 7.66 | 4.76 ± 6.70 |  |
| MG | 2.85 ± 2.55 | 2.53 ± 1.43 | 7.03 ± 10.94 | 7.84 ± 10.92 | 0.14 (-0.32, 0.59) |
| AG | 2.19 ± 1.64 | 4.88 ± 5.61 | 4.16 ± 5.48 | 3.10 ± 1.75 |  |
| **T** | 0.5 ± 0.23 | 0.65 ± 0.38 | 0.53 ± 0.33 | 0.63 ± 0.37 |  |
| MG | 0.47 ± 0.20 | 0.72 ± 0.54 | 0.61 ± 0.39 | 0.62 ± 0.49 | 0.04 (-0.18, 0.26) |
| AG | 0.51 ± 0.26 | 0.62 ± 0.29 | 0.48 ± 0.29 | 0.63 ± 0.31 |  |
| **T/C ratio** | 0.95 ± 3.12 | 1.73 ± 5.86 | 0.23 ± 0.18 | 0.27 ± 0.21 |  |
| MG | 2.20 ± 5.29 | 0.45 ± 0.51 | 0.21 ± 0.14 | 1.0 ± 0.13 | 0.02 (-0.38, 0.34) |
| AG | 0.27 ± 0.23 | 2.42 ± 7.26 | 0.25 ± 0.20 | 0.31 ± 0.24 |  |
| **sFR** | 0.41 ± 0.24 | 0.42 ± 0.28 | 0.47 ± 0.16 | 0.49 ± 0.28 |  |
| MG | 0.46 ± 0.26 | 0.48 ± 0.36 | 0.51 ± 0.16 | 0.67 ± 0.35 | 0.10 (-0.13, 0.33) |
| AG | 0.38 ± 0.28 | 0.39 ± 0.23 | 0.45 ± 0.16 | 0.39 ± 0.19 |  |
| **Absolute sIgA** | 333.3 ± 150.8 | 335.2 ± 236.7 | 384.2 ± 198.7 | 407.4 ± 198.7 |  |
| MG | 294.3 ± 74.8 | 226.3 ± 95.7 | 336.8 ± 111.3 | 269.9 ± 65.7 ‡ | -0.14 (-0.29, 0.01) |
| AG | 354.3 ± 178.4 | 393.8 ± 271.1 | 409.7 ± 233.0 | 481.4 ± 222.7 |  |
| **sIgA SR** | 133.7 ± 92.8 | 115.6 ± 72.6 | 171.1 ± 100.3 | 189.5 ± 130.9^b^ |  |
| MG | 129.7 ± 54.3 | 87.6 ± 56.9 | 167.5 ± 65.6 | 176.3 ± 98.9 | -0.40 (-0.28, 0.20) |
| AG | 135.8 ± 110.2 | 130.7 ± 77.7 | 173.0 ± 117.4 | 196.7 ± 148.5 |  |

‡ Significant group difference (independent t-test, p < 0.05).

^b^ Significantly different from T2.

Abbreviations: MG - Maladapted group AG - Adapted group, C- cortisol measured in nmol/L; T – testosterone measured in nmol/L; T/C ratio – testosterone to cortisol ratio, sFR- salivary flow rate measured in mL/min; sIgA SR – salivary immunoglobulin A secretion rate measured in μg·ml/min; Absolute sIgA – salivary immunoglobulin A measured in μg/mL.
