# Supplementary material for: Evidence That Sleep Is an Indicator of Overtraining during the Competition Phase of Adolescent Sprinters
Source: J Sports Med (Hindawi Publ Corp). 2021 Apr 3;2021:6694547. doi: 10.1155/2021/6694547 (PMC8041504; doi:10.1155/2021/6694547)
Supplement: Supplementary Materials — Table S1: performance data for each sprinter across the competitive phase of the athletic season. Table S2: the biochemical response of sprinters corresponding to different phases of the athletic season. [file 6694547.f1.zip › 6694547.f1/Supplimentary Table 1 (1).docx]

Table S1: Official competition times converted to scores using the International Association of Athletics Federations (IAAF) scoring tables of athletics.

| Date | Jan 4 | Jan 11 | Jan 18 | Jan 25 | Feb 1 | Feb 8 | Feb 15 | Feb 22 | Mar 8 | Mar 24 | **% change** |
| --- | --- | --- | --- | --- | --- | --- | --- | --- | --- | --- | --- |
|  | **Pre-competitive phase** | | | | | | | **Competition phase** | | |  |
| **Code** | **DM1** | **DM2** | **DM3** | **DM4** | **DM5** |  | **DM6** |  | **M1** | **M2** |  |
| 2354 | 866 |  | 837 |  | 866 |  | **886** |  | **1002** | *Cancelled* | 13 |
| 1589 |  | 820 | **886** | 883 | 860 |  | 836 |  | **699** |  | **-21** |
| 2020 | **1095** |  | 768 |  |  |  | 860 |  | **1128** |  | 3 |
| 9874 | **963** |  |  |  |  |  | 916 |  | **957** |  | -1 |
| 1994 | 859 |  | **895** |  | 882 |  |  |  | **901** |  | 1 |
| 9000 | 859 |  | 889 |  | **895** |  |  |  | **901** |  | 1 |
| 7895 | **1002** |  | 764 |  | 800 |  | 796 |  | **795** |  | **-21** |
| 4569 | 723 |  | 700 |  | **800** |  | 792 |  | **856** |  | 7 |
| 3030 | **899** |  | 870 |  |  |  | 849 |  | **803** |  | **-11** |
| 1253 |  | 963 |  | 1007 |  |  | **1049** |  | **1112** |  | 6 |
| 2001 | 887 |  | 871 |  |  |  | **906** |  | **929** |  | 3 |
| 5098 | **885** |  | 818 |  | 867 |  |  |  | **923** |  | 4 |
| 2004 |  | 870 | 956 | 952 | 540 |  | **996** |  | **1042** |  | 5 |
| 5340 |  | 751 |  |  | 751 |  | **757** |  | **774** |  | 2 |
| 3005 |  | 830 | 831 |  | 745 |  | **860** |  | **850** |  | -1 |
| 1010 |  | 772 | 784 |  |  |  | **808** |  | **864** |  | 7 |
| 1900 |  | 890 | **999** | 970 |  |  | 850 |  | **709** |  | **-29** |
| 1061 |  | 852 | **890** | 816 |  |  | 800 |  | **760** |  | **-15** |
| 4054 | **850** |  | 846 |  |  |  | 799 |  | **716** |  | **-16** |
| 3456 | **800** |  | 769 |  |  |  | 750 |  | **718** |  | **-10** |

Abbreviation DM – Development meet; M – major competition

Note: M2 was cancelled due to the coronavirus pandemic. % change was calculated as the best performance time (IAAF score) during the precompetitive phase minus the best performance time (IAAF Score) during the competition phase divided by the best performance time (IAAF Score) during the competition phase.
